# The genome sequence of the Four-dotted Obscure, *Oegoconia quadripuncta *(Haworth 1829)

**DOI:** 10.12688/wellcomeopenres.19737.1

**Published:** 2023-07-26

**Authors:** Gavin R. Broad, Natural History Museum Genome Acquisition Lab, Darwin Tree of Life Barcoding collective, Wellcome Sanger Institute Tree of Life programme, Wellcome Sanger Institute Scientific Operations: DNA Pipelines collective, Tree of Life Core Informatics collective, Darwin Tree of Life Consortium

**Affiliations:** 1 Natural History Museum, London, England, UK

**Keywords:** Oegoconia quadripuncta, Four-dotted Obscure, genome sequence, chromosomal, Lepidoptera

## Abstract

We present a genome assembly from an individual male *Oegoconia quadripuncta* (the Four-dotted Obscure; Arthropoda; Insecta; Lepidoptera; Autostichidae). The genome sequence is 622.6 megabases in span. Most of the assembly is scaffolded into 20 chromosomal pseudomolecules, including the Z sex chromosome. The mitochondrial genome has also been assembled and is 15.39 kilobases in length.

## Species taxonomy

Eukaryota; Metazoa; Eumetazoa; Bilateria; Protostomia; Ecdysozoa; Panarthropoda; Arthropoda; Mandibulata; Pancrustacea; Hexapoda; Insecta; Dicondylia; Pterygota; Neoptera; Endopterygota; Amphiesmenoptera; Lepidoptera; Glossata; Neolepidoptera; Heteroneura; Ditrysia; Gelechioidea; Autostichidae; Symmocinae; *Oegoconia*; *Oegoconia quadripuncta* (Haworth 1829) (NCBI:txid347754).

## Background

*Oegoconia quadripuncta*, which has fairly recently been given the name ‘Four-dotted Obscure’, is one of only three native species of the family Autostichidae in Britain. Specimens of the genus *Oegoconia* are distinctive little moths, dark brown with usually three pale yellow stripes across the fore wings, which are held very flat. In Britain and Ireland these were all referred to as *Oegoconia quadripuncta* until Goddard (1966) recognised *O. deauratella* as occurring here, and then Agassiz (1982) demonstrated that *O. caradjai* Popescu-Gorj & Capuse was another previously overlooked resident. All three species are externally very similar and only reliably identified by examination of their genitalia. Genitalia are well illustrated by Sterling *et al.* (2012) and in resources such as the mothdissection.co.uk website. The sequenced specimen, a male, was identified from the dissected genitalia, particularly the shape of the saccus, confirmed by the COI barcode.

Adults of *O. quadripuncta* are frequent in light traps in the summer, found throughout much of England and Wales but only recorded locally in Ireland and not in Scotland ( Sterling *et al.*, 2012). Larvae feed on decaying leaves in the litter layer, often below trees and hedgerows, as do the larvae of *O. caradjai* Popescu-Gorj & Capuse and, it is presumed, *O. deauratella*. In Britain, *O. quadripuncta* is invariably described as the most frequently collected species of *Oegoconia* and is the only one of the three which the first author has found at a couple of regularly sampled sites, but in Belgium it seems that *O. caradjai* Popescu-Gorj & Capuse is the most common and *O. quadripuncta* rarely found ( De Prins, 2005).

Autostichidae are part of the species-rich superfamily Gelechioidea ( Wang & Li, 2020), for which very few genome assemblies are currently available, with no previous Autostichidae genome. The fourth species on the British list, *Symmoca signatella* Herrich-Schäffer, is very different in appearance and probably an occasional accidental import. Interestingly, there are no reports of parasitoid wasps attacking *Oegoconia*, or any Autostichidae, although some must do so.

## Genome sequence report

**Figure 1.**
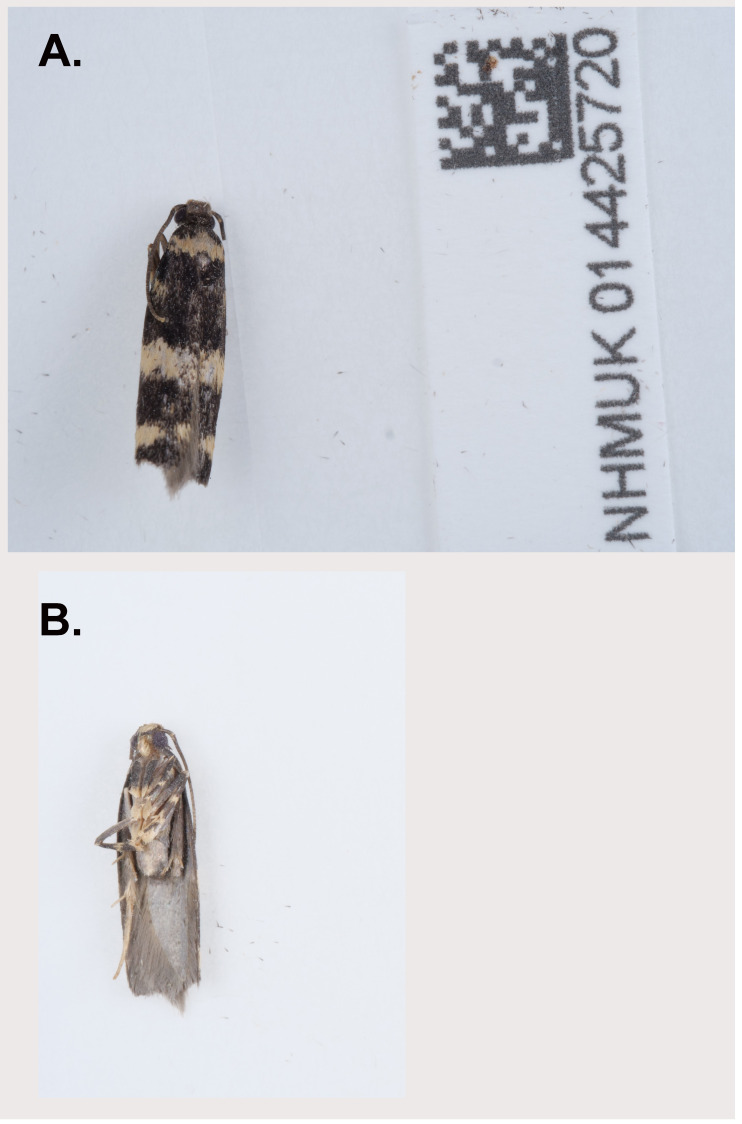
Photograph of the *Oegoconia quadripuncta* (ilOegQuad1) specimen used for genome sequencing. **A.** Dorsal view, **B.** Ventral view.

The genome was sequenced from one male *Oegoconia quadripuncta* ( Figure 1) collected from Tonbridge, Kent (51.19, 0.29). A total of 46-fold coverage in Pacific Biosciences single-molecule HiFi long reads was generated. Primary assembly contigs were scaffolded with chromosome conformation Hi-C data. Manual assembly curation corrected 25 missing joins or misjoins and removed 10 haplotypic duplications, reducing the assembly length by 0.35% and the scaffold number by 4.26%.

**Figure 2.**
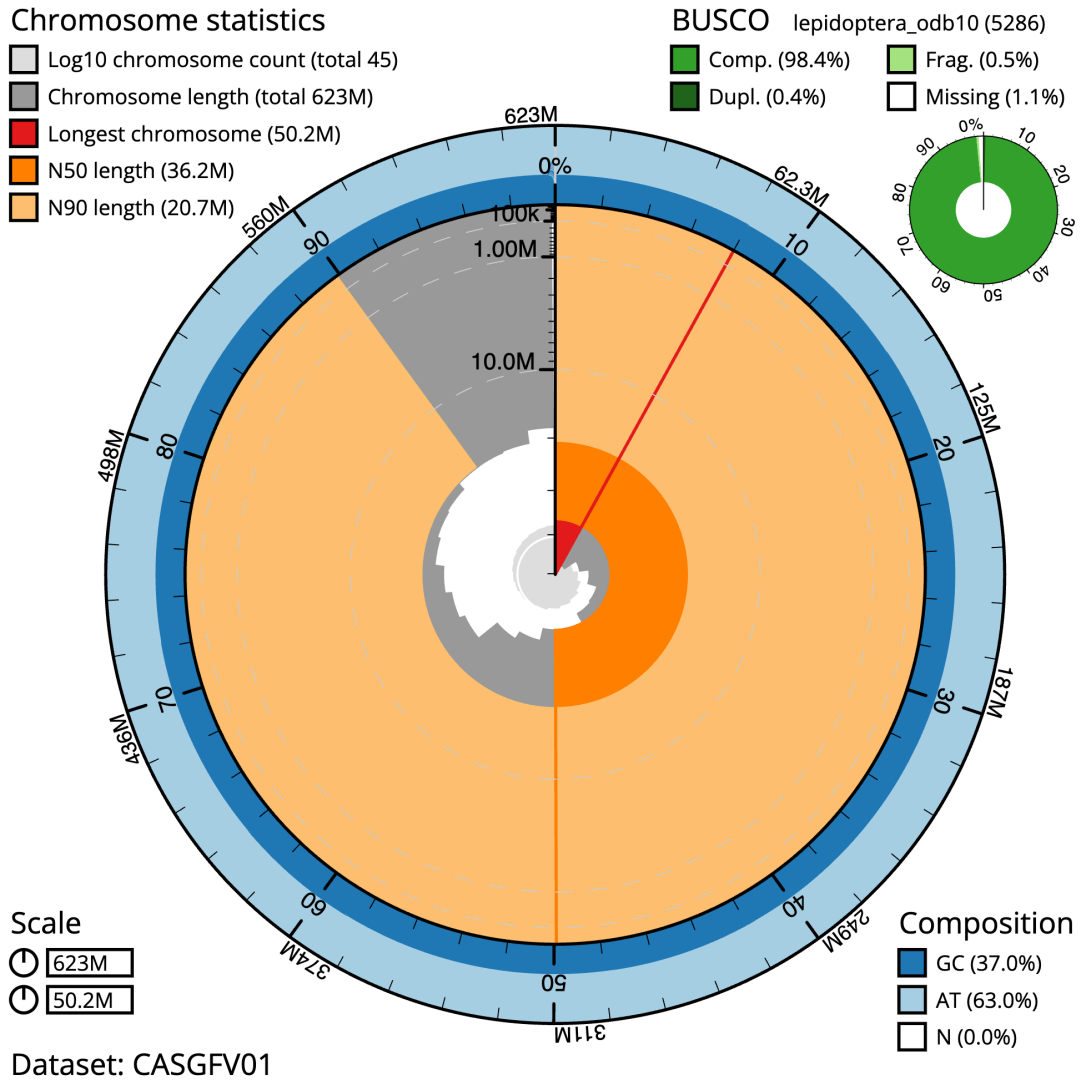
Genome assembly of *Oegoconia quadripuncta*, ilOegQuad1.1: metrics. The BlobToolKit Snailplot shows N50 metrics and BUSCO gene completeness. The main plot is divided into 1,000 size-ordered bins around the circumference with each bin representing 0.1% of the 622,656,778 bp assembly. The distribution of scaffold lengths is shown in dark grey with the plot radius scaled to the longest scaffold present in the assembly (50,213,132 bp, shown in red). Orange and pale-orange arcs show the N50 and N90 scaffold lengths (36,190,098 and 20,651,493 bp), respectively. The pale grey spiral shows the cumulative scaffold count on a log scale with white scale lines showing successive orders of magnitude. The blue and pale-blue area around the outside of the plot shows the distribution of GC, AT and N percentages in the same bins as the inner plot. A summary of complete, fragmented, duplicated and missing BUSCO genes in the lepidoptera_odb10 set is shown in the top right. An interactive version of this figure is available at https://blobtoolkit.genomehubs.org/view/ilOegQuad1.1/dataset/CASGFV01/snail.

The final assembly has a total length of 622.6 Mb in 44 sequence scaffolds with a scaffold N50 of 36.2 Mb ( Table 1). Most (99.75%) of the assembly sequence was assigned to 20 chromosomal-level scaffolds, representing 19 autosomes and the Z sex chromosome. Chromosome-scale scaffolds confirmed by the Hi-C data are named in order of size ( Figure 2– Figure 5; Table 2). While not fully phased, the assembly deposited is of one haplotype. Contigs corresponding to the second haplotype have also been deposited. The mitochondrial genome was also assembled and can be found as a contig within the multifasta file of the genome submission.

**Table 1.** Genome data for *Oegoconia quadripuncta*, ilOegQuad1.1.

Project accession data		
Assembly identifier	ilOegQuad1.1	
Species	*Oegoconia quadripuncta*	
Specimen	ilOegQuad1	
NCBI taxonomy ID	347754	
BioProject	PRJEB59195	
BioSample ID	SAMEA111458706	
Isolate information	ilOegQuad1, male: whole organism (DNA sequencing and Hi-C scaffolding)	
Assembly metrics *		*Benchmark*
Consensus quality (QV)	62.5	*≥ 50*
*k*-mer completeness	100%	*≥ 95%*
BUSCO **	C:98.4%[S:98.0%,D:0.4%],F:0.5%,M:1.1%,n:5,286	*C ≥ 95%*
Percentage of assembly mapped to chromosomes	99.75%	*≥ 95%*
Sex chromosomes	Z chromosome	*localised homologous pairs*
Organelles	Mitochondrial genome assembled	*complete single alleles*
Raw data accessions		
PacificBiosciences SEQUEL II	ERR10812851	
Hi-C Illumina	ERR10818304	
Genome assembly		
Assembly accession	GCA_949316235.1	
*Accession of alternate * *haplotype*	GCA_949316225.1	
Span (Mb)	622.6	
Number of contigs	177	
Contig N50 length (Mb)	6.7	
Number of scaffolds	44	
Scaffold N50 length (Mb)	36.2	
Longest scaffold (Mb)	50.2	

* Assembly metric benchmarks are adapted from column VGP-2020 of “Table 1: Proposed standards and metrics for defining genome assembly quality” from ( Rhie *et al.*, 2021). ** BUSCO scores based on the lepidoptera_odb10 BUSCO set using v5.3.2. C = complete [S = single copy, D = duplicated], F = fragmented, M = missing, n = number of orthologues in comparison. A full set of BUSCO scores is available at https://blobtoolkit.genomehubs.org/view/ilOegQuad1.1/dataset/CASGFV01/busco.

**Figure 3.**
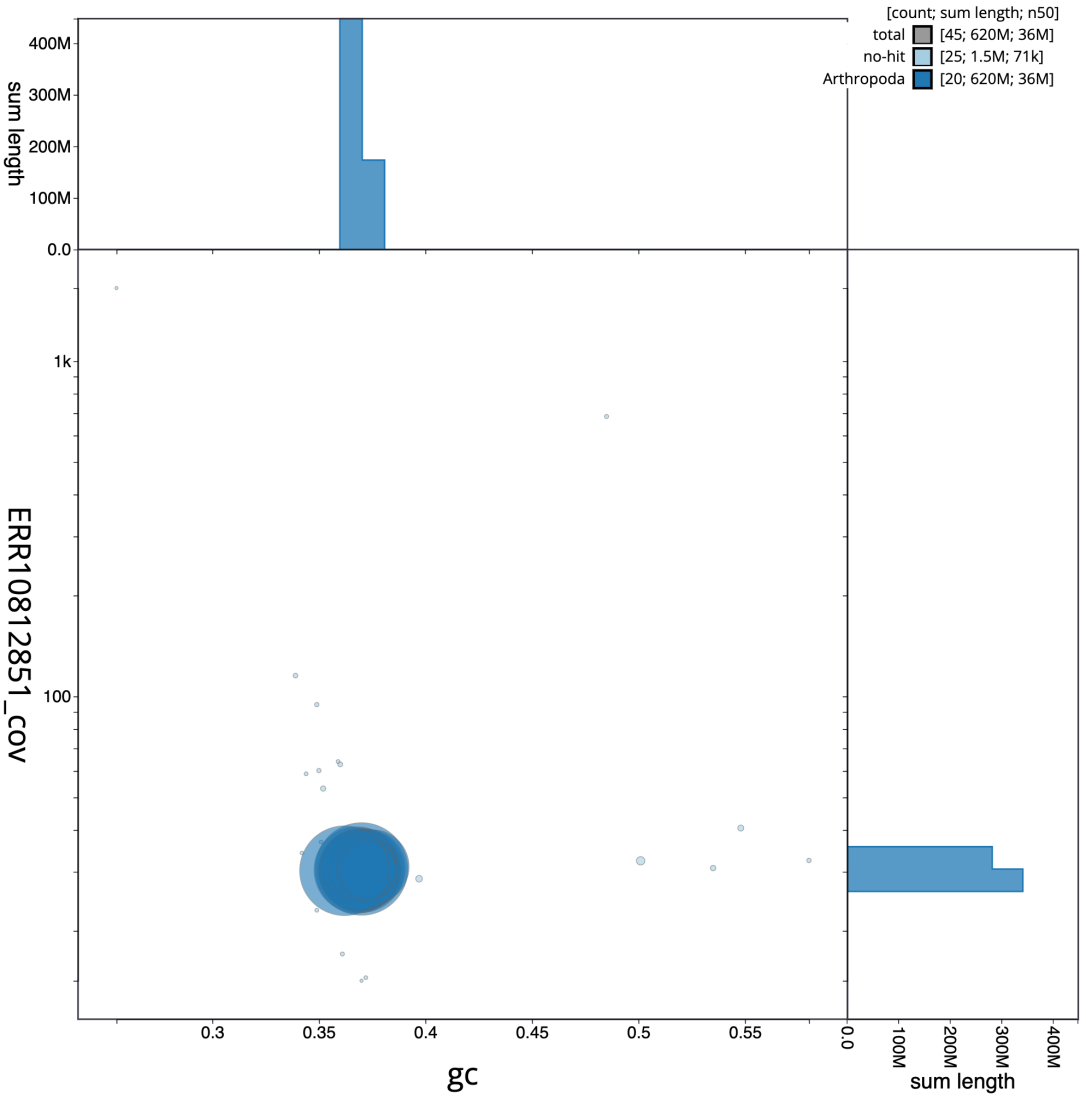
Genome assembly of *Oegoconia quadripuncta*, ilOegQuad1.1: BlobToolKit GC-coverage plot. Scaffolds are coloured by phylum. Circles are sized in proportion to scaffold length. Histograms show the distribution of scaffold length sum along each axis. An interactive version of this figure is available at https://blobtoolkit.genomehubs.org/view/ilOegQuad1.1/dataset/CASGFV01/blob.

**Figure 4.**
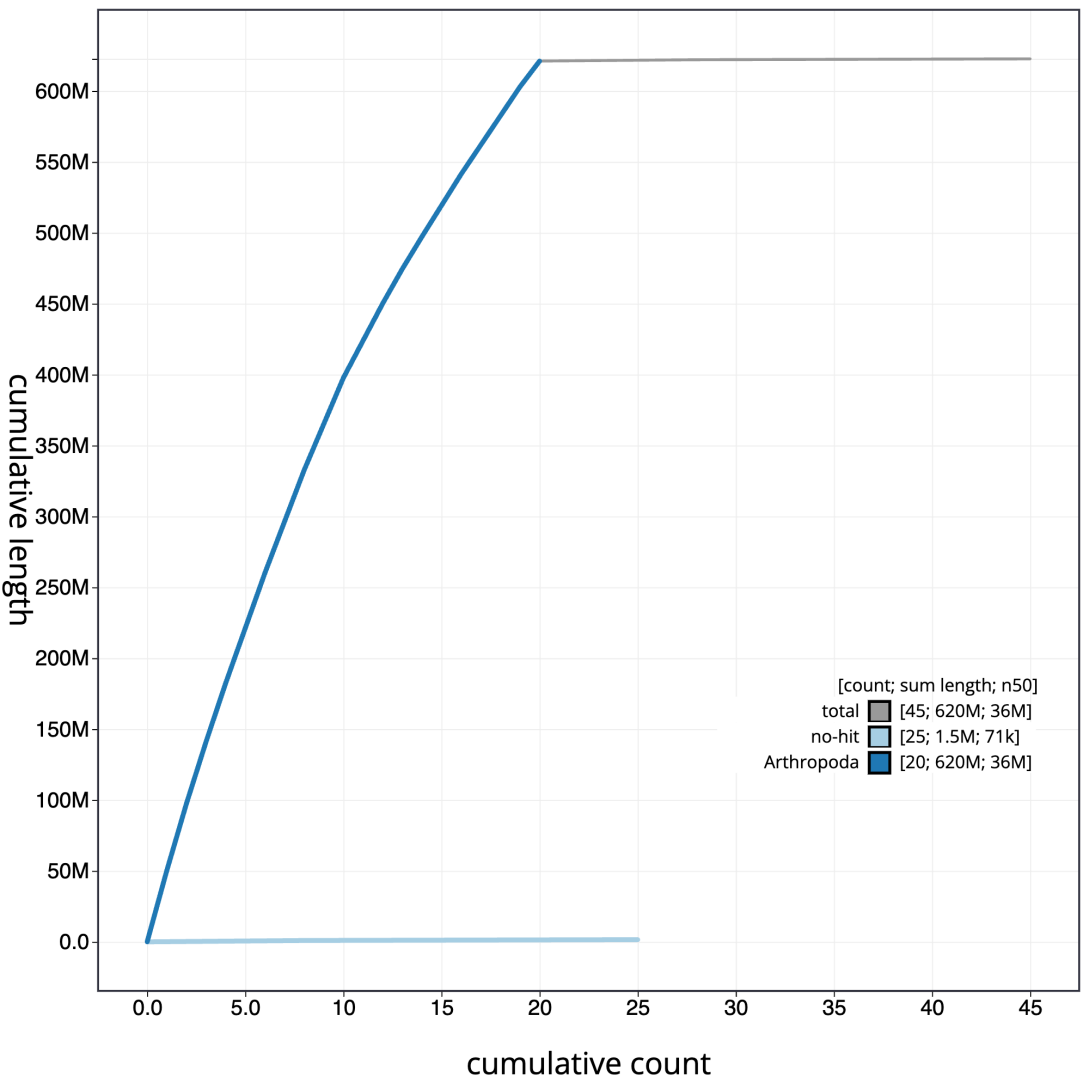
Genome assembly of *Oegoconia quadripuncta*, ilOegQuad1.1: BlobToolKit cumulative sequence plot. The grey line shows cumulative length for all scaffolds. Coloured lines show cumulative lengths of scaffolds assigned to each phylum using the buscogenes taxrule. An interactive version of this figure is available at https://blobtoolkit.genomehubs.org/view/ilOegQuad1.1/dataset/CASGFV01/cumulative.

**Figure 5.**
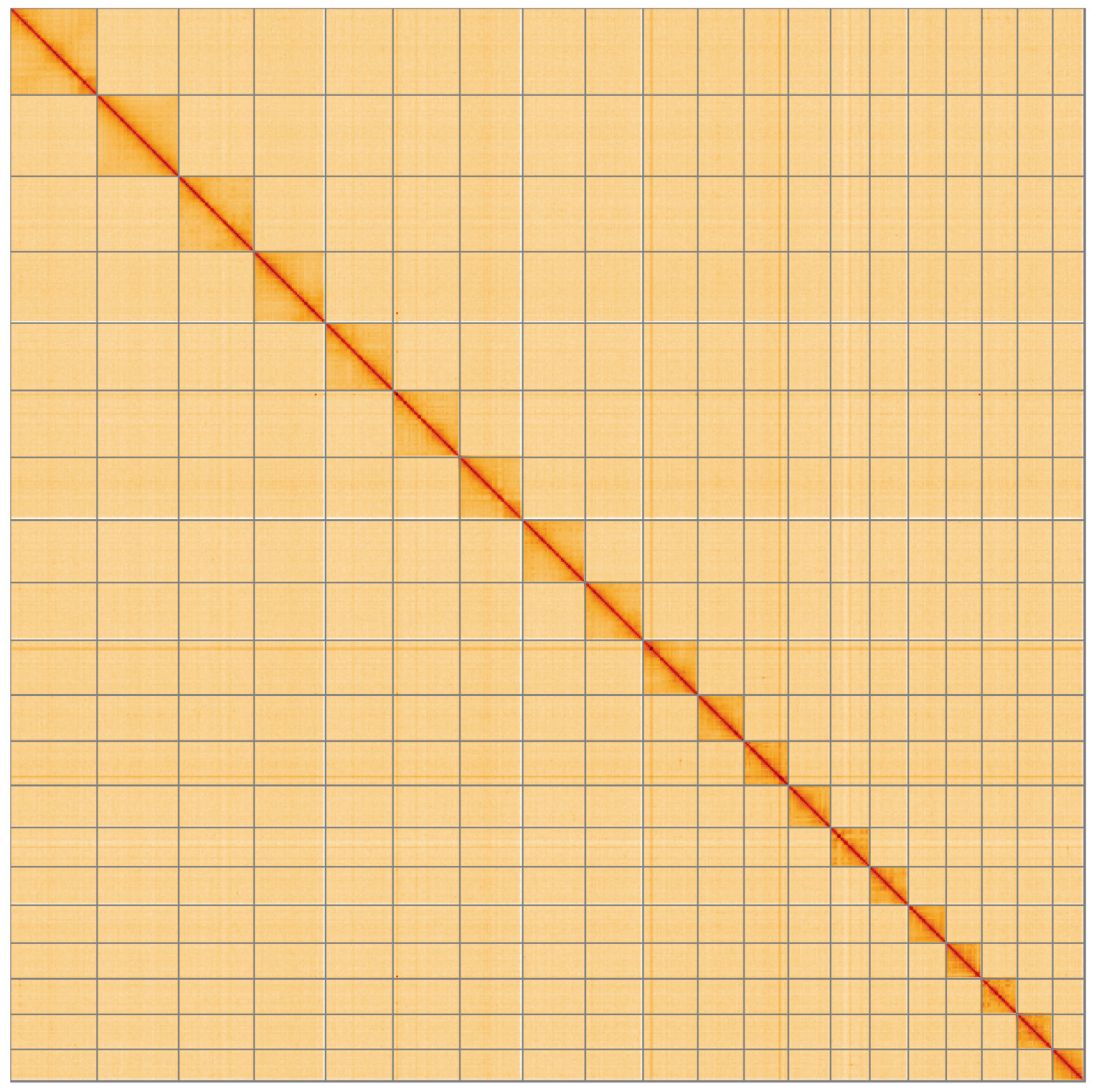
Genome assembly of *Oegoconia quadripuncta*, ilOegQuad1.1: Hi-C contact map of the ilOegQuad1.1 assembly, visualised using HiGlass. Chromosomes are shown in order of size from left to right and top to bottom. An interactive version of this figure may be viewed at https://genome-note-higlass.tol.sanger.ac.uk/l/?d=WvXNmCQqSuiNwWWPD2TKSg.

**Table 2.** Chromosomal pseudomolecules in the genome assembly of *Oegoconia quadripuncta*, ilOegQuad1.

INSDC accession	Chromosome	Length (Mb)	GC%
OX438741.1	1	50.21	37.0
OX438743.1	2	43.64	37.0
OX438744.1	3	41.32	37.0
OX438745.1	4	39.03	37.0
OX438746.1	5	38.66	37.0
OX438747.1	6	36.36	37.0
OX438748.1	7	36.19	37.0
OX438749.1	8	33.42	37.0
OX438750.1	9	31.67	37.5
OX438751.1	10	26.73	37.0
OX438752.1	11	25.63	37.0
OX438753.1	12	24.4	36.5
OX438754.1	13	22.74	36.5
OX438755.1	14	22.2	37.0
OX438756.1	15	21.93	37.0
OX438757.1	16	20.65	37.0
OX438758.1	17	20.61	37.0
OX438759.1	18	20.38	37.5
OX438760.1	19	18.16	37.5
OX438742.1	Z	47.17	36.0
OX438761.1	MT	0.02	25.5

The estimated Quality Value (QV) of the final assembly is 62.5 with *k*-mer completeness of 100%, and the assembly has a BUSCO v5.3.2 completeness of 98.4% (single = 98.0%, duplicated = 0.4%), using the lepidoptera_odb10 reference set ( *n* = 5,286).

Metadata for specimens, spectral estimates, sequencing runs, contaminants and pre-curation assembly statistics can be found at https://links.tol.sanger.ac.uk/species/347754.

## Methods

### Sample acquisition and nucleic acid extraction

A male *Oegoconia quadripuncta* (specimen ID NHMUK014425720, ToLID ilOegQuad1) was collected using a light trap from a garden in Tonbridge, Kent, UK (latitude 51.19, longitude 0.29) on 2021-08-05. The specimen was collected and identified by Gavin Broad (Natural History Museum) and dry-frozen at –80°C.

The sample was prepared for DNA extraction at the Tree of Life laboratory, Wellcome Sanger Institute (WSI). The ilOegQuad1 sample was weighed and dissected on dry ice with tissue set aside for Hi-C sequencing. Tissue from the whole organism was disrupted using a Nippi Powermasher fitted with a BioMasher pestle. DNA was extracted at the WSI Scientific Operations core using the Qiagen MagAttract HMW DNA kit, according to the manufacturer’s instructions.

### Sequencing

Pacific Biosciences HiFi circular consensus DNA sequencing libraries were constructed according to the manufacturers’ instructions. DNA sequencing was performed by the Scientific Operations core at the WSI on a Pacific Biosciences SEQUEL II (HiFi) instrument. Hi-C data were also generated from tissue of ilOegQuad1 using the Arima2 kit and sequenced on the Illumina NovaSeq 6000 instrument.

### Genome assembly, curation and evaluation

Assembly was carried out with Hifiasm ( Cheng *et al.*, 2021) and haplotypic duplication was identified and removed with purge_dups ( Guan *et al.*, 2020). The assembly was then scaffolded with Hi-C data ( Rao *et al.*, 2014) using YaHS ( Zhou *et al.*, 2023). The assembly was checked for contamination and corrected using the gEVAL system ( Chow *et al.*, 2016) as described previously ( Howe *et al.*, 2021). Manual curation was performed using gEVAL, HiGlass ( Kerpedjiev *et al.*, 2018) and Pretext ( Harry, 2022). The mitochondrial genome was assembled using MitoHiFi ( Uliano-Silva *et al.*, 2022), which runs MitoFinder ( Allio *et al.*, 2020) or MITOS ( Bernt *et al.*, 2013) and uses these annotations to select the final mitochondrial contig and to ensure the general quality of the sequence.

A Hi-C map for the final assembly was produced using bwa-mem2 ( Vasimuddin *et al.*, 2019) in the Cooler file format ( Abdennur & Mirny, 2020). To assess the assembly metrics, the *k*-mer completeness and QV consensus quality values were calculated in Merqury ( Rhie *et al.*, 2020). This work was done using Nextflow ( Di Tommaso *et al.*, 2017) DSL2 pipelines “sanger-tol/readmapping” ( Surana *et al.*, 2023a) and “sanger-tol/genomenote” ( Surana *et al.*, 2023b). The genome was analysed within the BlobToolKit environment ( Challis *et al.*, 2020) and BUSCO scores ( Manni *et al.*, 2021; Simão *et al.*, 2015) were calculated.

Table 3 contains a list of relevant software tool versions and sources.

**Table 3.** Software tools: versions and sources.

Software tool	Version	Source
BlobToolKit	4.1.5	https://github.com/blobtoolkit/blobtoolkit
BUSCO	5.3.2	https://gitlab.com/ezlab/busco
gEVAL	-	https://geval.org.uk/
Hifiasm	0.16.1-r375	https://github.com/chhylp123/hifiasm
HiGlass	1.11.6	https://github.com/higlass/higlass
Merqury	MerquryFK	https://github.com/thegenemyers/MERQURY.FK
MitoHiFi	2	https://github.com/marcelauliano/MitoHiFi
PretextView	0.2	https://github.com/wtsi-hpag/PretextView
purge_dups	1.2.3	https://github.com/dfguan/purge_dups
sanger-tol/ genomenote	v1.0	https://github.com/sanger-tol/genomenote
sanger-tol/ readmapping	1.1.0	https://github.com/sanger-tol/readmapping/tree/1.1.0
YaHS	1.2a	https://github.com/c-zhou/yahs

### Wellcome Sanger Institute – Legal and Governance

The materials that have contributed to this genome note have been supplied by a Darwin Tree of Life Partner. The submission of materials by a Darwin Tree of Life Partner is subject to the **‘Darwin Tree of Life Project Sampling Code of Practice’**, which can be found in full on the Darwin Tree of Life website here. By agreeing with and signing up to the Sampling Code of Practice, the Darwin Tree of Life Partner agrees they will meet the legal and ethical requirements and standards set out within this document in respect of all samples acquired for, and supplied to, the Darwin Tree of Life Project.

Further, the Wellcome Sanger Institute employs a process whereby due diligence is carried out proportionate to the nature of the materials themselves, and the circumstances under which they have been/are to be collected and provided for use. The purpose of this is to address and mitigate any potential legal and/or ethical implications of receipt and use of the materials as part of the research project, and to ensure that in doing so we align with best practice wherever possible. The overarching areas of consideration are:

•   Ethical review of provenance and sourcing of the material

•   Legality of collection, transfer and use (national and international)

Each transfer of samples is further undertaken according to a Research Collaboration Agreement or Material Transfer Agreement entered into by the Darwin Tree of Life Partner, Genome Research Limited (operating as the Wellcome Sanger Institute), and in some circumstances other Darwin Tree of Life collaborators.

## Data Availability

European Nucleotide Archive: *Oegoconia quadripuncta*. Accession number PRJEB59195; https://identifiers.org/ena.embl/PRJEB59195. ( Wellcome Sanger Institute, 2023) The genome sequence is released openly for reuse. The *Oegoconia quadripuncta* genome sequencing initiative is part of the Darwin Tree of Life (DToL) project. All raw sequence data and the assembly have been deposited in INSDC databases. The genome will be annotated using available RNA-Seq data and presented through the Ensembl pipeline at the European Bioinformatics Institute. Raw data and assembly accession identifiers are reported in Table 1.

## Author information

Members of the Natural History Museum Genome Acquisition Lab are listed here: https://doi.org/10.5281/zenodo.4790042.

Members of the Darwin Tree of Life Barcoding collective are listed here: https://doi.org/10.5281/zenodo.4893703.

Members of the Wellcome Sanger Institute Tree of Life programme are listed here: https://doi.org/10.5281/zenodo.4783585.

Members of Wellcome Sanger Institute Scientific Operations: DNA Pipelines collective are listed here: https://doi.org/10.5281/zenodo.4790455.

Members of the Tree of Life Core Informatics collective are listed here: https://doi.org/10.5281/zenodo.5013541.

Members of the Darwin Tree of Life Consortium are listed here: https://doi.org/10.5281/zenodo.4783558.
