# Supplementary material for: The genome sequence of the Four-dotted Obscure, Oegoconia quadripuncta (Haworth 1829)
Source: Wellcome Open Res. 2023 Jul 26;8:328. [Version 1] doi: 10.12688/wellcomeopenres.19737.1 (PMC11369589; doi:10.12688/wellcomeopenres.19737.1)
Supplement: Peer review history

# Reviewer report: Reviewer response for version 1

**DOI:** 10.21956/wellcomeopenres.21861.r96658

**Published:** 2024-09-02

**Authors:** Juan Wulff

**Affiliations:** 1 North Carolina State University, Raleigh, USA

- “The sequenced specimen, a male, was identified from the dissected genitalia, particularly the shape of the saccus, confirmed by the COI barcode” Please move this to Methods section.
- First time other species are mentioned in the manuscript, *e.g*. O. *quadripuncta,* the complete species’ name should be given.
- For future reports, using a scale bar in the pictures could be useful to see the actual specimens’ size.
- It may be useful for future studies if the DNA of bacteria and other microorganisms is provided separately from the genome assembly but all together with an associated ID within the same Bioproject.
- Gene annotation should be improved in future studies.

Are sufficient details of methods and materials provided to allow replication by others?

Yes

Is the rationale for creating the dataset(s) clearly described?

Yes

Are the datasets clearly presented in a useable and accessible format?

Yes

Are the protocols appropriate and is the work technically sound?

Yes

Reviewer Expertise:

Entomology, Molecular biology and Genetics

I confirm that I have read this submission and believe that I have an appropriate level of expertise to confirm that it is of an acceptable scientific standard.

# Reviewer report: Reviewer response for version 1

**DOI:** 10.21956/wellcomeopenres.21861.r89670

**Published:** 2024-08-21

**Authors:** Daniel Bruzzese

**Affiliations:** 1 Yale University, New Haven, Connecticut, USA

The authors present the 622 Mb genome assembly of *Oegoconia quadripuncta*. The genome is of excellent near-chromosomal quality and is mostly localized on 20 chromosomes. This assembly is an important contribution as there are currently very few genomes from the diverse and understudied Gelechoidea.

 I have no major comments and I believe this paper and dataset are ready for indexing.

 My only minor comment would be to provide some clarification to the background section. In the third paragraph please clarify that *Symmoca signatella* is probably non-native. I was surprised that suddenly a fourth moth appeared when I was only expecting three.

Are sufficient details of methods and materials provided to allow replication by others?

Yes

Is the rationale for creating the dataset(s) clearly described?

Yes

Are the datasets clearly presented in a useable and accessible format?

Yes

Are the protocols appropriate and is the work technically sound?

Yes

Reviewer Expertise:

Evolutionary biology, genomics, phylogenomics, symbiosis, Rhagoletis, tsetse flies, and trypanosomes.

I confirm that I have read this submission and believe that I have an appropriate level of expertise to confirm that it is of an acceptable scientific standard.
